# Supplementary material for: The mediating role of green innovation and green culture in the relationship between green human resource management and environmental performance
Source: PLoS One. 2022 Sep 29;17(9):e0274820. doi: 10.1371/journal.pone.0274820 (PMC9521903; doi:10.1371/journal.pone.0274820)
Supplement: Attachment — Submitted filename: Response to Reviewers.docx [file pone.0274820.s001.docx]

**Response to reviewer's comments**

Dear editor and reviewers,

Many thanks for your academic guidance on our manuscript entitled "**The Mediating Role of Green Innovation and Green Culture in the Relationship between Green Human Resource Management and Environmental Performance**" (ID: PONE-D-22-00680). All the suggestions are objective, comprehensive, professional, and meticulous. We have studied carefully and substantially revised our original manuscript after reading the comments provided by the two reviewers. Besides, the revised parts are marked **in red** for your review. We have copy-pasted your comments and followed them with our responses for your and the reviewers' convenience.

**Response to Reviewer #1:**

**Comments:**

Expand literature background (and deriving assumptions for the study) by including a more in-depth investigation of related topics such as CSR (Corporate Social Responsibility) and circular economy/business models.

Illustrate better the nature (more than just the composition) of the sample and explain better how results can be generalized or not (you mention in the limitations section but need to be more specific on why the paper is anyhow a contribution to academic/practitioner discussion).

Enhance discussion with a more robust and in-depth investigation of where/how the study advances current knowledge, also at the light of the research gap identified in the introduction.
Have the paper proofread by a mother tongue.

**Response:**

Thank you very much for your positive comments on our work.

In the literature and hypotheses section, we expanded the literature and discussed our study with related topics, including CSR.

Santana, Morales-Sánchez, and Pasamar's (2020) studies in human resources have shown how essential it is to have an effective H.R. management for a business to achieve its goals successfully. Therefore, when the firm's social responsibility is outlined in economic, social, and environmental goals, H.R. management should be focused toward accomplishing those objectives to fulfill the organization's social obligation. A recent study showed that GHRM affects environmental performance and plays a significant role in how corporate social responsibility (CSR) affects environmental performance (Kraus et al., 2020).

Researchers focus on HRM and emphasize the strategic contributions of HRM, particularly innovation, new product creation, and the management of talent (Smith, 2018). According to the findings of research conducted by Farouk et al. (2016), HRM has a significant impact on innovation and has a direct influence on innovation. Another research on socially responsible human resource management (SRHRM), green innovation, and the environmental performance of Bangladesh's banking industry indicated that SRHRM had a positive influence on green innovation (Rakin, Yousuf & Rubel, 2020).

Imran and Jingzu (2022) conducted an extensive study on green innovation and environmental performance as a moderator. The study's findings show that environmental performance moderates the link between green innovation and organizational performance.

In addition, the research that has been done in the past demonstrates that the cultural emphasis on environmental performance is focused on green HRM and corporate social responsibility. As a result of this, we examine and evaluate existing literature that has already researched the fields of corporate social responsibility (CSR) and green culture with organizational environmental performance (Kraus et al., 2020; Chuang & Huang, 2018), as well as the development of green culture over time (Ogiemwonyi, & Harun, 2021). Given that a dedication to the environment is widely recognized as an incontestable and innate quality of culture strategies, we focus a significant portion of our attention on the topic of environmentally conscious culture.

**Response:**

We have further elaborated on the nature of the sample and sought to explain better how the results of this study could be generalized.

We have enhanced the discussion (in the revised manuscript) of how this study furthers existing knowledge in the light of the research gap identified in the introduction with more robust and in-depth research.

The results shed light on the fact that sustainable human resource management considerably boosts environmental performance. The results confirmed that Jabbar and Abid (2015) found that green HRM can boost an organization's overall performance. The GHRM has a considerable impact on the green culture. The results are consistent with those found by Muisyo et al. (2021), which state that GHRM is an essential component in the development of green culture. It has been established in the research that GHRM is very reliant on the creativity of the business. GHRM is often seen as a forward-thinking endeavor inside organizations (Renkema, Meijerink, & Bondarouk, 2021). The findings are consistent with Song et al. (2020). In addition, the findings demonstrate that the degree to which green culture influences environmental performance is considerable. The findings are consistent with those of Roscoe et al. (2019) in that a green culture provides the way for businesses to acquire environmental competencies that ultimately result in a competitive advantage.

Meanwhile, green innovations make a substantial contribution to improved environmental performance. The results are similar to those found by Imran and Jingzu (2022), which indicate that green innovations significantly increase the performance of organizations. In conclusion, the relationship between GHRM and environmental performance is considerably mediated by green innovation and green culture. According to the findings of this research, GHRM has both a direct and an indirect effect on the environment's performance.

We have provided proofread certificate (in a supplementary file). An expert has proofread this article.

**Response to Reviewer** #**2:**

**Comments**

More explanation of methodology analysis needed. For example, what model it was used, which software, what types of tests were conducted and why? I believe the limitation section need to include the low level of AVE and why you do think this low level appears and how future studies could try resolve that.

**Response:**

We have updated the manuscript based on the suggestions of a respected reviewer.

In the present research, the suggested hypotheses were put through an empirical test with the help of SmartPLS 3.3.7. The utilization of SmartPLS 3.3.7 is recommended because it provides successful results, is capable of managing simple as well as complicated or extensive research models and does not need the performance of normality tests (Sarstedt, & Cheah, 2019; Hair Jr, Hult, Ringle, & Sarstedt, 2014). In addition, the findings of some earlier studies indicate that the PLS-SEM approach is better than the covariance-based technique in terms of the quality of the data it provides (Rigdon, Sarstedt, & Ringle, 2017; Dash, & Paul, 2021). In PLS-SEM, the estimation process used two models, including the measurement and structural models. In this study, we employed both of the models that have been previously mentioned.

**Measurement model**

It is essential to calculate three main tests when evaluating a measurement model: content validity, convergent validity, and discriminant validity (Hair Jr et al., 2014).

**Convergent validity**

Convergent validity is how variables' items measure the same variable. (Ayoup, Rehman, & Mohamed, 2019). According to Imran et al. (2021), convergent validity evaluates if all constructs' items accurately represent their accompanied predictor. Convergent validity was assessed to identify two techniques: average variance extracted (AVE) and composite reliability (CR). Additionally, factor loadings, AVE, and CR values should be greater than 0.50, 0.50, and 0.60, respectively (Hair et al., 2013). Cronbach's alpha value should be more than 0.60, according to Nunnally (1978). In Table 2, it can be shown that the AVE has values of more than 0.50, and the CR value is greater than 0.60, as mentioned by Hair et al. (2013). Furthermore, Cronbach's alpha value is larger than 0.60, higher than the 0.60 indicated by Nunnally (1978).

**Discriminant validity**

According to statistical definitions, discriminant validity is when a study evaluates two statistically distinct elements (Imran et al., 2021). To evaluate discriminant validity, first, compute the square roots of AVE and then compare them to the correlations of both of these variables in the theoretical model (Chin, 2010). Furthermore, the diagonal values of comprised constructs should be higher than the values in the same rows and with columns in both rows and columns (Fornell and Larcker, 1981). As we can see, Table 3 illustrates that the present research meets the criteria for discriminant validity. Fornell and Larcker (1981) proposed that any diagonal upper values more than other related values in the same columns and rows are shown in Table 2, which is also included in the following Table 3.

**Content validity**

Content validity is defined as the degree to which elements of a measurement instrument are relevant to and characteristic of the targeted construct for a specific assessment objective (Cook, & Beckman, 2006). In this study, the content validity is evaluated with the guidance of factor loadings, which are shown in Figure 2 of cross-loadings. This technique was recommended by several earlier studies (Chin, 1998). Each item of the study's relevant variables is heavily loaded regarding this particular item of study.

**Limitations and future research**

Despite the research contributions and implications, this study has several limitations that might be addressed by other research in the future. First, this research used a cross-sectional approach. The researchers are unsure whether or not GHRM, green innovation, and green culture in manufacturing organizations provide similar impacts over a longer period. In light of this, upcoming researchers might use the same study model to determine whether or not the results of their investigations contradict. The data for this study originated from the Malaysian manufacturing sector. Still, researchers can also collect data from small and medium-sized enterprises to compare the two sets of findings in the future. In addition, the GHRM, green innovation, and green culture are used to ascertain environmental performance for this research. To determine in the future whether or not it is relevant, the researchers can employ green capacity and CSR as mediating variables between GHRM and environmental performance. In conclusion, this study was carried out within the context of Malaysia, which is reflective of a certain culture. On the other hand, future researchers expand on this, and they show that the same research model can be performed in emerging countries and developed nations to examine how the results differ.

**Comment:**

What ethical procedures were followed?

**Response**:

To fulfil the requirements of the procedures for data collection, we have sent official university letters to employees in the manufacturing sector. These letters reference the Personal Data Protection Act of 2010. ("PDPA") of Malaysia.

All that we have revised is marked **in red** in our manuscript.

We hope to have addressed your concerns on the mentioned issues. Thank you very much again for your helpful comments, which we greatly appreciate. We welcome any additional comments that you may have.
